# Nasal irrigation with various solutions for adults with allergic rhinitis: A protocol for systematic review and meta-analysis of randomized controlled trials

**DOI:** 10.1097/MD.0000000000031884

**Published:** 2022-11-25

**Authors:** Asti Widuri, Bambang Udji Djoko Rianto, Luh Putu Lusy Indrawati, Ranggaputra Nugraha, Abdul Wahab

**Affiliations:** a Doctoral program in Faculty of Medicine, Public Health and Nursing, Universitas Gadjah Mada, Yogyakarta, Indonesia; b Departement of Otorhinolaryngology Head and Neck Surgery, Faculty of Medicine and Health Sciences, Universitas Muhammadiyah, Yogyakarta, Indonesia; c Departement of Otorhinolaryngology Head and Neck Surgery, Faculty of Medicine, Public Health and Nursing, Universitas Gadjah Mada, Yogyakarta, Indonesia; d Departement of Biostatistics Epidemiology and Population Health, Faculty of Medicine, Public Health and Nursing, Universitas Gadjah Mada, Yogyakarta, Indonesia.

**Keywords:** allergic rhinitis, nasal irrigation, solutions, therapy

## Abstract

Nasal douching or irrigation has been recommended for adjunctive therapy of sinonasal diseases including allergic rhinitis (AR) for many years. Previous study reported large-volume high-pressure device as an effective standard application, but the solutions was remains controversy. This study systematically review the clinical efficacy of nasal irrigation with various solutions for adults with AR from medical literature.

**Methods:**

This research systematically asses clinical trial about nasal irrigation with various solutions for adults with AR from medical literature. The sources were PubMed, ProQuest, Scopus, Cochrane Register of Controlled Trials databases, and gray literature from google scholar and RAMA repository limited to English and Bahasa Indonesia language articles, published from January 2017 to July 2022. Only randomized controlled trials involving the human subjects studies will be included. The inclusion criteria research must be related to nasal irrigation for AR, and should be full texted available. Literature management, screening, data extraction will use Rayyan.ai tools. The quality assessment of qualified paper and risk of bias will be assessing independent conducted by 2 reviewer with risk of bias 2. We will use Review Manager (RevMan) [Computer program] Version 5.4. The Cochrane Collaboration, 2020 tools to produce the systematic review and meta-analysis.

**Results:**

After completion of the study process, the data analysis and review will be reported. The results will be publicized through a peer-review journal publication.

**Conclusion:**

The results of the systematic review will summarize the efficacy of various nasal irrigation for adults with AR, so it can be used as clinician recommendation.

## 1. Introduction

### 1.1. Description of the condition

Allergic Rhinitis (AR) is a common health problem worldwide that affects almost all ages whose prevalence varies among populations and locations.^[1]^ The prevalence of AR has increased drastically developing countries, due to genetic predisposition, epigenetic events, and changes in environmental exposure.^[2,3]^ AR is a chronic inflammation of the nasal mucosa caused by an allergic reaction, with the main symptoms of rhinorrhea, nasal obstruction, sneezing, and pruritus.^[4]^ Symptoms that last a long time in productive age cause morbidity due to physical and psychological effects on quality of life.^[5]^

When inhaling allergens and sticking to the nasal mucosa, someone with a sensitive immune system will stimulate Immunoglobulin E production, mediated inflammatory response. Impaired epithelial barrier function has been hypothesized to contribute to allergic reactions through increased antigenic pathways and exposure of underlying tissues to these stimuli.^[6,7]^ Pharmacologic therapy, such as intranasal corticosteroids (INCS), non-sedating antihistamines (AH), decongestants, and non-pharmacological strategies to avoid allergens, are used to treat AR.^[8,9]^

### 1.2. Description of the intervention

Nasal irrigation is an ancient traditional practice recommended as adjuvant treatment for AR.^[10]^ It can promote mucociliary clearance as a host defense and protection mechanism by increasing the mechanical clearance of nasal mucosa’s secretions, allergens, and inflammatory mediators.^[11]^ Nasal irrigation also reduces allergen-mediated proteolytic activity and inflammatory cytokines that prevent disruption of the sinonasal epithelial barrier.

### 1.3. How the intervention might work

AR treatment that is effective for a long time is expensive and has potential side effects such as sedation and irritation of the nasal mucosa. Some AR patients prefer non-pharmacological therapy, such as nasal irrigation and medicinal preparations, to protect the nasal mucosa from allergen exposure. A recent review study concluded that isotonic saline nasal irrigation as a complementary therapy in AR results in improved symptoms, quality of life, mucociliary clearance and reduced drug consumption.^[10]^ For sinonasal diseases, hypertonic solutions improve symptoms than isotonic solutions but the minor side effects are more signigicant.^[12]^ Hypertonic saline may be more effective than isotonic in treating pediatric AR.^[13]^

### 1.4. The importance of review

Nasal saline irrigation is effective as adjuvant therapy for AR. The method is relatively easy, inexpensive, and has few side effects. However, in patients with persistent severe symptoms, the role of nasal saline irrigation is limited. Therefore, several attempts have an idea to combine or add saline solution with anti-inflammatory, immunomodulatory, anti-allergic properties.

This systematic review focused on the objective in assessing and synthesizing the clinical effectiveness of nasal irrigation with various solutions for adults with AR.

## 2. Methods and design

The Preferred Reporting Items Guidelines for Systematic Review and Meta-Analysis Protocols (PRISMA-P) were used to conduct this systematic review protocol.^[14]^ This protocol has been submitted to the International Prospective Register of Systematic Review with the registration number CRD42022330598, and may find at https://www.crd.york.ac.uk/PROSPERO/display_record.php?RecordID=330598.

The process of creating this systematic review will dividd into 5 stages (preparation, retrieval, assessment, synthesis, and writing). The task follows 15 steps: formulating review questions, finding previous systematic reviews, writing protocols, developing search strategies, search, de-duplicate, abstract screen, obtain full text, filter full text, snowball, extract data, synthesize data, reexamine literature, meta-analysis and writing a review.^[15]^

### 2.1. Criteria of study include in this review

#### 2.1.1. Type of study.

Only prospective randomized controlled studies (RCTs) of nasal irrigation therapy for adults (≥18 years old) with AR disease will include. The eligible language is limited to English and Bahasa Indonesia.

#### 2.1.2. Types of participants.

All adult patients diagnosed with AR from history, physical examination and positive result from at least 1 of laboratory characteristic.

#### 2.1..3. Types of interventions.

Only papers with interventions involving nasal irrigation as primary therapy used alone or in conjunction with standard drug therapy were included. Each consignment method (wash, irrigation, douche, spray, or nebulizer), various devices (neti pot, squeeze bottle, syringe, pump, nebulizer), any volume and frequency or duration will be included.

The solutions compositions for nasal irrigation are as follows:

1. Saline solution with varied tonicity and alkalinity concentrations.
2. Solution containing ions.
3. Adding a matrix involved in barrier protection, enforcing wound healing, and mucosal surface repair or herbal with biologically active molecules enhance the efficacy of the nasal solution.

#### 2.1.4. Types of comparators.

The comparator included standard conventional medication therapy, placebo or saline solution.

#### 2.1.5. Types of outcomes measures.

The primary outcome include the clinical effectiveness and quantitative assessment disease severity of specific symptoms such as Total Nasal Symptom Score, Visual Analogue Scales and adverse effects. The secondary outcomes are the quality of life impact of certain diseases including Sino-Nasal Outcome Test-22, mucocilliary function and serum inflammatory markers.

### 2.2. Exclusion criteria

#### 2.2.1. The exclusion criteria for this review.

(1) Patients with nasal tumors and autoimmune-mediated diseases.
(2) Patient with sinusitis dental complication.
(3) Chronic rhinosinusitis patients with or without nasal polyps.

### 2.3. Search strategy for identification of studies

Search literature on this study topic from January 2017 to June 2022 through Cochrane Library, Scopus, PubMed, ProQuest databases. MESH (Medical Subject Headings) keywords and terms to capture all relevant articles. Eligible studies were selected based on inclusion criteria. The combination of MESH terms and keywords is (“rhinitis, allergy” [Mesh Terms] OR AR [Text Word] OR “Perennial AR” OR “Seasonal AR”) AND ((“rinse nose”[Mesh Terms] OR nasal irrigation [Word Text] OR “nasal irrigation” OR “saline irrigation” OR “sinus irrigation” OR “nose rinse” OR “saline nasal wash” OR “nose rinse”).

### 2.4. Searching other resources

We scanned the reference lists of eligible studies, also previous systematic reviews relevant to this topic for additional trials. The nonsystematic searches (hand searches) were also run of the Google Scholar and RAMA repositories to retrieve gray literature and other potential trials.

### 2.5. Data collection and analysis

#### 2.5.1. Study selection.

**Figure 1.**
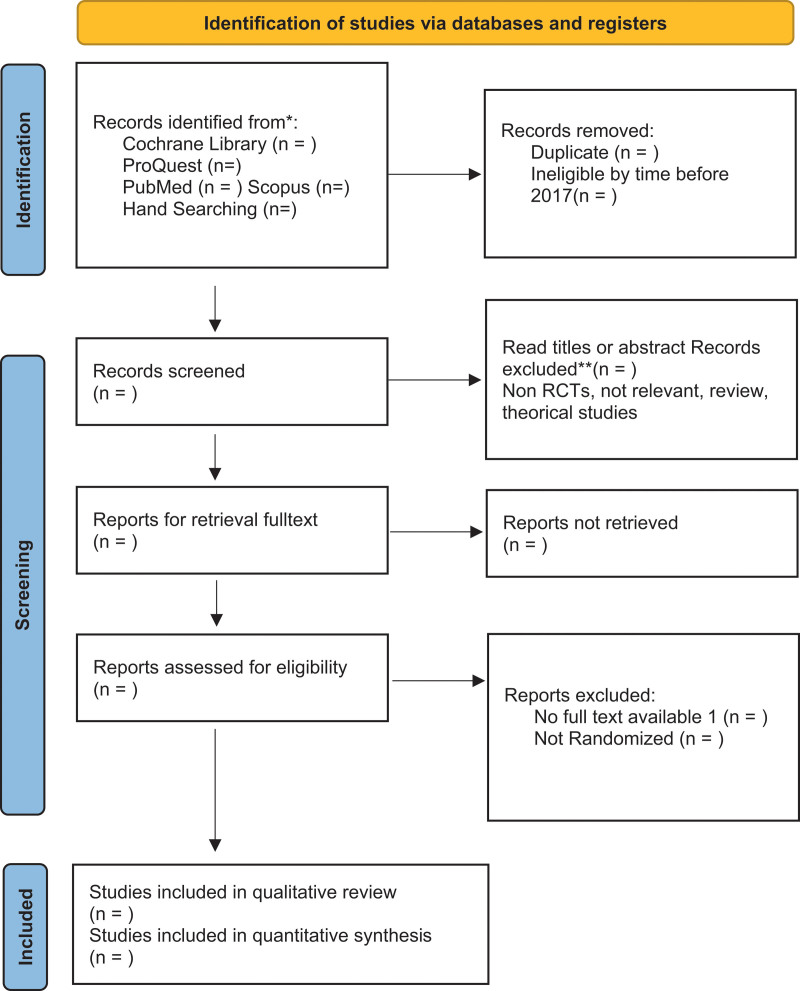
A flow chart for new systematic review retrieval and selection.

The study will be identify by 2 independent reviewers, data management using Rayyan.ai for de-duplicate and abstract assignments and title screens. The process will be documented with the PRISMA 2020 flowchart (Fig. 1) and the full text obtained after being issued for any reason was extracted and analyzed using Revman 5.4.

#### 2.5.2. Data extraction and management.

Both reviewers (AWA and RN) conducted independent searches with exact keywords. All articles obtained from that search strategy were imported into Mendeley to eliminate duplicate studies. Two independent researchers concerning inclusion criteria screened abstracts and titles. After that, we analyzed the filtered full texts retrospectively. Finally, we organized the included articles in an excel spreadsheet file. The discussion between the 2 reviewers and a consultant will be do if any discrepancies and the 2 have not agreed.

#### 2.5.3. A quality assessment of included studies.

Studies selected that met the inclusion criteria were assessed methodologically by 2 reviewers (AWA and RN) using the standard ROB 2 instrument for RCTs at the study selection stage. Discussions are held to resolve any assessment disagreements between 2 reviewers or to consult with a third reviewer (BUD) if necessary.

#### 2.5.4. Measures of treatment effect.

For each trial, the description is as follow:

The study characteristic are the year, author, number of arms with intervention, and comparison/placebo. The following are the characteristics of trial participants: mean age and standard deviation, gender (% of male and female), and the total number of patients included in the analysis. Type of administration, device, volume, duration of nasal irrigation.

The outcome measures as stated above.

A tabulation will summarize these data.

#### 2.5.5. Problem analysis analysis.

Studies that do not use randomization will not be used (right or left nasal side analysis as a unit).

#### 2.5.6. Missing data solution.

If possible, we ask the author to request the missing data. We associate missing data with surrogate values and treat them as observed. We make explicit assumptions about any methods used to address missing data, perform sensitivity analysis, and resolve issues with potential impact on discovery.

#### 2.5.7. Assessment of heterogeneity.

We assessed variability between studies (intervention effects evaluated), recognizing uncertainty in measures such as *I*^*2*^ when multiple studies exist.

#### 2.5.8. Reporting bias assessment.

##### 2.5.8.1. Outcome reporting bias.

The mitigation strategies to minimize potential bias followed the systematic review guideline in reporting such as grading of recommendation assessment, development, and evaluation (GRADE).

##### 2.5.8.2. Publication bias.

A comprehensive literature search across 4 source databases and searching the gray literature/unpublished literature are the strategy to minimize the effect of bias.^[16]^

### 2.6. Data synthesis

#### 2.6.1. Subgroup analysis.

We performed subgroup analyses for efficacy, quality of life, and side effects, side effects, to explore differences between patients with intervention and controls risk of bias. Two investigators (AW and RN) independently assessed trial study quality, addressing randomization, allocation concealment, blinding of observers and participants, outcome completeness, selective reporting and other potential sources of bias. Odds ratios (for categorical outcome data), standardized mean differences (for continuous data) and 95% confidence intervals will calculated from the data. Generated by each randomized controlled trial included as quantitative data Revman 5.4 application to extract data.

#### 2.6.2. Sensitivity analysis.

A sensitivity analysis is a repeat of the primary analysis with changes to the data or changes in which the data analysis. We will use a sensitivity analysis to investigate whether our review’s findings depend on decisions made during the review process. We can concluded that the results are robust if from the sensitivity analysis shows that the findings are not dependent on these decisions and uncertainties in the data.

#### 2.6.3. Confidence in cumulative evidence.

GRADE (Grading of Recommendation Assessment, Development, and Evaluation) is a method for assessing the overall certainty (quality) of evidence, which reflects how certain we are that the effect estimates are valid and sufficient to support a therapeutic choice or recommendation.^[17]^ The GRADE scale for cumulative evidence has 4 levels: high, moderate, low, and very low. The determinant factor approach assigns a high quality rating to data from RCTs with no major constraints and a low-quality rating to observational studies. Five domains that can lead to the downgrading certainty were limitation in detail study design (risk of bias), inconsistency, indirectness, imprecision and publication factors. The large amplitude of impact, opposing apparent residual bias or confounding, and dose-response gradient are 3 domains that can boost assurance.^[18]^

## 3. Discussion

This protocol outlines a systematic review and meta-analysis planed and conducted to determine the efficacy of nasal irrigation with various solutions in adults with AR. Previous comprehensive evaluations have demonstrated the effectiveness of saline solution in reducing nasal symptoms without the adding materials to repair epithelial and nasal mucosa damage. The strategy for treating AR includes allergen avoidance, second-generation AH, and INCS. A combination of AH/INCS should be investigated in patients who do not react to INCS. Allergen immunotherapy is an option for people who do not respond to or do not want to take pharmacotherapy for a long time.^[19]^

Intranasal administration of drugs for local and systemic delivery also has several potentials for AR treatment. Due to its noninvasive nature, large mucosa surface area, fast onset absorption and avoidance of first-pass metabolism. INCS, AH, decongestants, and nasal irrigation are all recommended in international guidelines for AR treatment. However, the drugs, drug excipients such as preservatives, absorption enhancers, and various nasal irrigation solutions may affect the nasal mucociliary function.^[20]^

## Acknowledgements

The authors thank the reviewers for their feedback.

## Author contributions

AW started the project, the protocol was constructed by AW, AW, and RN. Each study’s search approach, screening, and critical assessment are carried out independently by AW and RN. AW^d^ would oversee subgroup and sensitivity analyses. BUD and LPI oversaw and revised all stages of the manuscript while AW drafted it. The final manuscript was read and approved by all of the authors.

**Conceptualization:** Asti Widuri, Bambang Udji Djoko Rianto, Luh Putu Lusy Indrawati, Ranggaputra Nugraha, Abdul Wahab.

**Formal analysis:** Abdul Wahab.

**Methodology:** Ranggaputra Nugraha.

**Supervision:** Bambang Udji Djoko Rianto, Luh Putu Lusy Indrawati.

**Writing – original draft:** Asti Widuri.

**Writing – review & editing:** Asti Widuri, Ranggaputra Nugraha.
